# Stimuli-responsive electrospun nanofibers for drug delivery, cancer therapy, wound dressing, and tissue engineering

**DOI:** 10.1186/s12951-023-01987-z

**Published:** 2023-07-24

**Authors:** Kai Chen, Yonghui Li, Youbin Li, Yinfeng Tan, Yingshuo Liu, Weisan Pan, Guoxin Tan

**Affiliations:** 1 Key Laboratory of Tropical Translational Medicine of Ministry of Education, Hainan provincial key laboratory of R&D on tropical herbs, Haikou Key Laboratory of Li Nationality Medicine, School of Pharmacy, Hainan Medical University, Haikou, 571199 People’s Republic of China; 2 Key Laboratory of Tropical Biological Resources of Ministry of Education, School of Pharmacy, Hainan University, Haikou, 570228 People’s Republic of China; 3 School of Pharmacy, Shenyang Pharmaceutical University, Shenyang, 110016 People’s Republic of China

**Keywords:** Stimuli-responsive, Nanofibers, Electrospinning, Drug delivery, Biomedicine

## Abstract

The stimuli-responsive nanofibers prepared by electrospinning have become an ideal stimuli-responsive material due to their large specific surface area and porosity, which can respond extremely quickly to external environmental incitement. As an intelligent drug delivery platform, stimuli-responsive nanofibers can efficiently load drugs and then be stimulated by specific conditions (light, temperature, magnetic field, ultrasound, pH or ROS, etc.) to achieve slow, on-demand or targeted release, showing great potential in areas such as drug delivery, tumor therapy, wound dressing, and tissue engineering. Therefore, this paper reviews the recent trends of stimuli-responsive electrospun nanofibers as intelligent drug delivery platforms in the field of biomedicine.

## Introduction

Stimuli-responsive materials are a new class of intelligent materials developed based on the concept of bionics, which can respond to small changes in the environment, such as temperature, pH, magnetic field, light, and ultrasound, through significant changes in their chemical or physical properties [1, 2]. Due to their functional properties, stimuli-responsive materials have a wide range of applications in the field of biomedicine [3–5]. Researchers have developed a large number of stimuli-response materials, including hydrogels [6], microspheres [7], micelles [8], and nanofibers [9], to enhance the efficacy of drug treatment or to confer special biological functions [10]. The stimuli-responsive hydrogels with bulk structures respond slowly to external stimuli, because the large size of the hydrogels makes it difficult for external stimuli to diffuse throughout the materials. Although microspheres and micelles dispersed in water in the form of nanoparticles have a fast stimulus response speed, their application in living organisms is limited due to their limited stability and lack of macroscopic shape and mechanical strength. Nanofiber membranes formed by random or ordered arrangement of nanofibers not only have macroscopic shape and mechanical strength, but also respond quickly to external stimuli, so they have broader application prospects in the field of biomedicine [11, 12].

Electrospinning can prepare polymer fibers nanometer diameter, and possesses the advantages of simple operation, extensive application range and high production efficiency [13, 14]. Electrospun nanofibers have high specific surface area, diverse structures, wide sources of preparation materials, unique physicochemical properties, and flexibility in surface modification [15, 16]. In addition, electrospun nanofibers can mimic the structure of the extracellular matrix (ECM), which can promote cell adhesion, proliferation, differentiation, and guide tissue repair and regeneration [17, 18]. These unique properties appealed to researchers to endow electrospun nanofibers with the ability to respond to stimuli. Stimuli-responsive electrospun nanofibers have extremely fast response speed to external stimuli due to their large specific surface area and porosity [19]. More importantly, they can efficiently load drugs, thereby realizing active drug release in vivo by controlling changes in environmental conditions [20]. In addition, the preparation materials of stimuli-responsive electrospun nanofibers have a wide range of sources, and the production process is simple and fast, which is promising to solve the problem of scale-up production of stimuli-responsive materials [21].

**Scheme 1:**
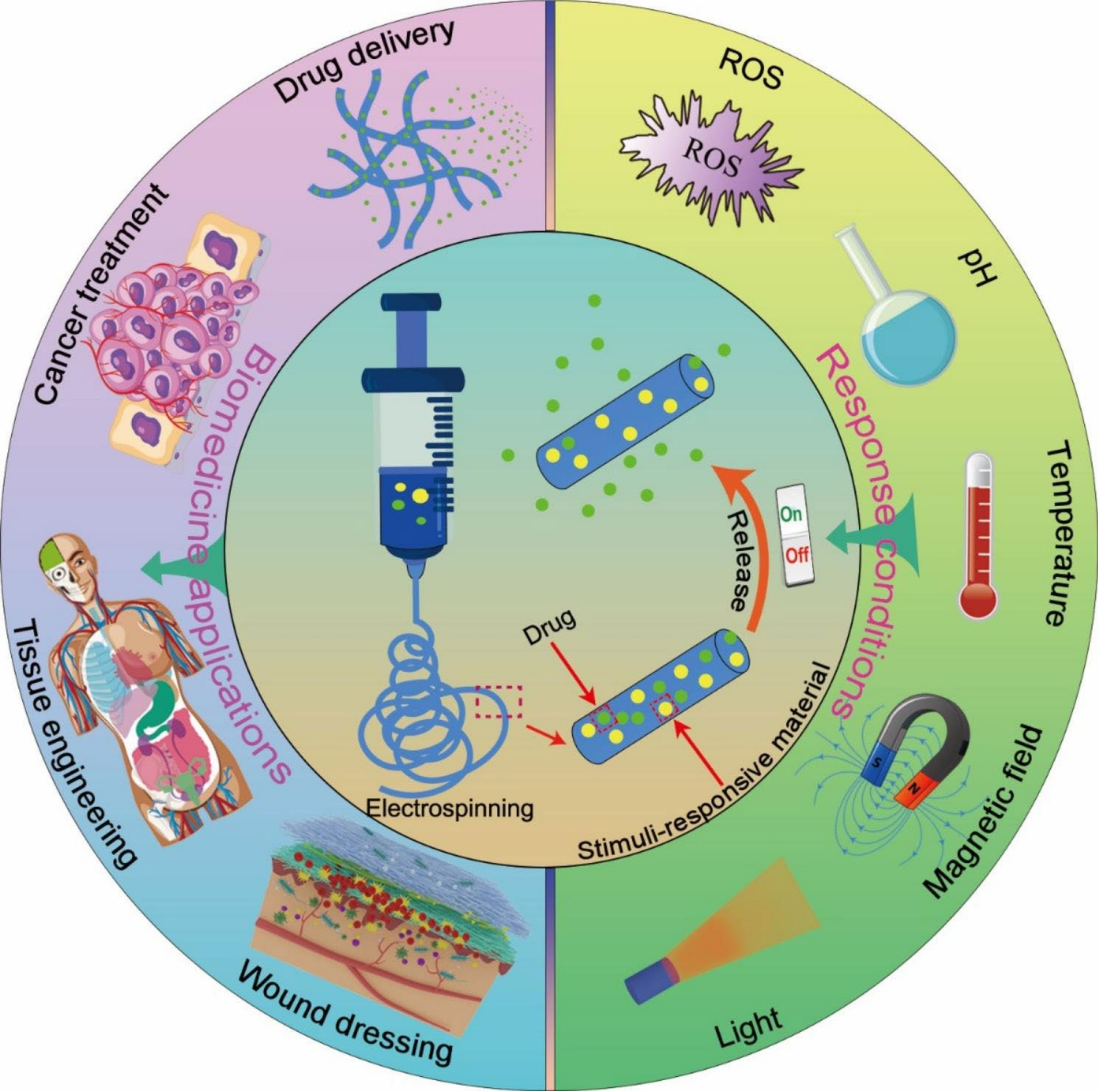
Schematic illustration of stimuli-responsive electrospun nanofibers as intelligent drug delivery platform in biomedicine field

Therefore, this paper reviews the recent trends of intelligent drug delivery systems constructed from stimuli-responsive electrospun nanofibers in the biomedical field (Scheme 1). Firstly, the preparation methods of stimuli-response electrospun nanofibers were summarized. Then the design strategies of various types of stimuli-responsive electrospun nanofibers were discussed. In addition, the research progress of stimuli-responsive electrospun nanofibers in drug delivery, tumor therapy, wound dressing and tissue regeneration were introduced. Finally, the challenges and future development of stimuli-responsive electrospun nanofibers in clinical application were presented.

## Preparation method of stimuli-responsive electrospun nanofibers

### Principle of electrospinning

**Fig. 1:**
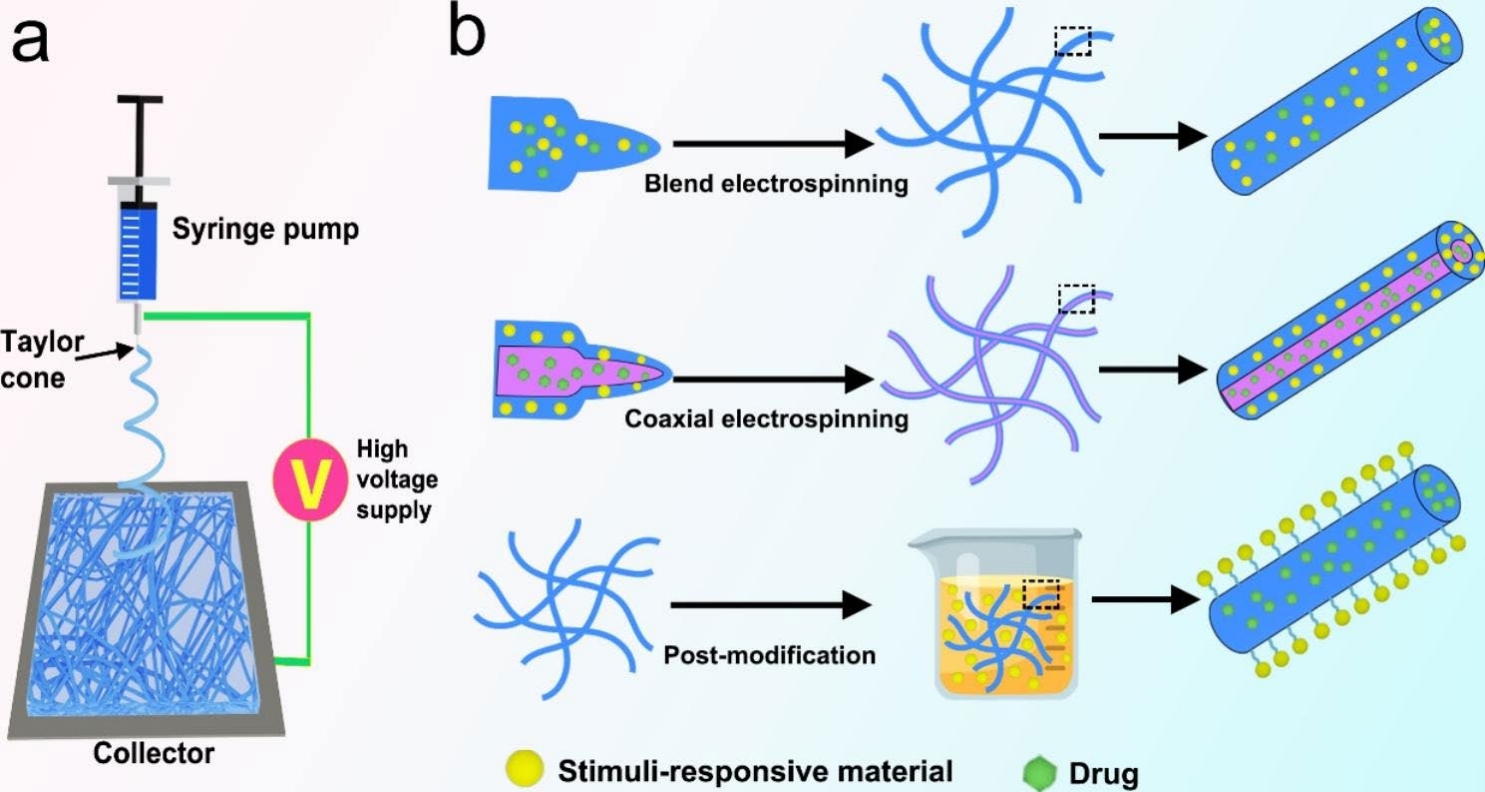
(**a**) Schematic of a conventional electrospinning equipment. (**b**) Schematic illustration of three common methods for preparing stimuli-responsive electrospun nanofibers

Electrospinning is a process in which the charged polymer solution is stretched and deformed by the electric field force generated by high-voltage static electricity, and then the nanofibers are obtained by volatilization of the solvent [22, 23]. The electrospinning equipment is mainly composed of high voltage power supply, syringe pump and collector (Fig. 1a) [24]. During the spinning process, the spinning liquid droplets in the syringe pump are subjected to two opposite forces: surface tension and electric field force. As the electric field force increases, the droplet is stretched into a cone shape, called a Taylor cone [25]. The droplet is sprayed from the tip of the Taylor cone onto the collector when the electric field force overcomes the surface tension of the droplet. The jet is stretched and lengthened, and the solvent is volatilized continuously [26]. Finally, the jet solidifies on the collector to form nanofibers [27]. The electrospinning process is controlled by numerous parameters, mainly involving solution parameters (e.g., viscosity, concentration, conductivity of the spinning solution, and evaporation rate of the solvent), process parameters (e.g., applied voltage, a flow rate of the polymer solution, and collection distance), and environmental parameters (temperature and humidity) [28, 29].

### Materials and solvents

The materials used to prepare nanofibers by electrospinning are generally polymers. At present, more than 100 kinds of polymers have been successfully prepared as nanofibers, which can be divided into synthetic polymers and natural polymers. Synthetic polymers such as polyvinyl alcohol (PVA), polycaprolactone (PCL), polylactic acid (PLA), polyethylene oxide (PEO), polyurethane (PU) and other materials have controllable mechanical strength and physical properties, and excellent spinning performance [30, 31]. Due to the absence of biological properties of synthetic polymers, natural polymers are more appealing as biomedical materials. Natural polymers such as collagen, silk fibroin, gelatin, chitosan (CS), and hyaluronic acid (HA) have good biocompatibility and biodegradability, and can be better recognized by cell surface receptors in the process of tissue repair, thus triggering cell adhesion and proliferation [32]. However, the spinnability and mechanical properties of a single natural polymer are poor, so many researchers have developed natural/synthetic polymer composite fibers to take into account the advantages of two different polymers at the same time, so as to have better performance [33].

Common solvents utilized in electrospinning include formic acid, dichloromethane, hexafluoroisopropanol (HFIP), dimethylformamide (DMF), acetic acid, ethanol, trifluoroacetic acid, tetrahydrofuran and distilled water [34]. The properties of the solvent such as the dielectric constant, conductivity, volatility, and solubility of the polymer will have a significant effect on the electrospinning process and the morphology of nanofibers [35]. The effect of spinning with a single solvent is often not ideal, notably for the preparation of composite nanofibers. Therefore, it is frequently essential to use blend solvents to improve spinnability and nanofiber morphology.

### Preparation methods of stimuli-responsive electrospun nanofibers

**Fig. 2:**
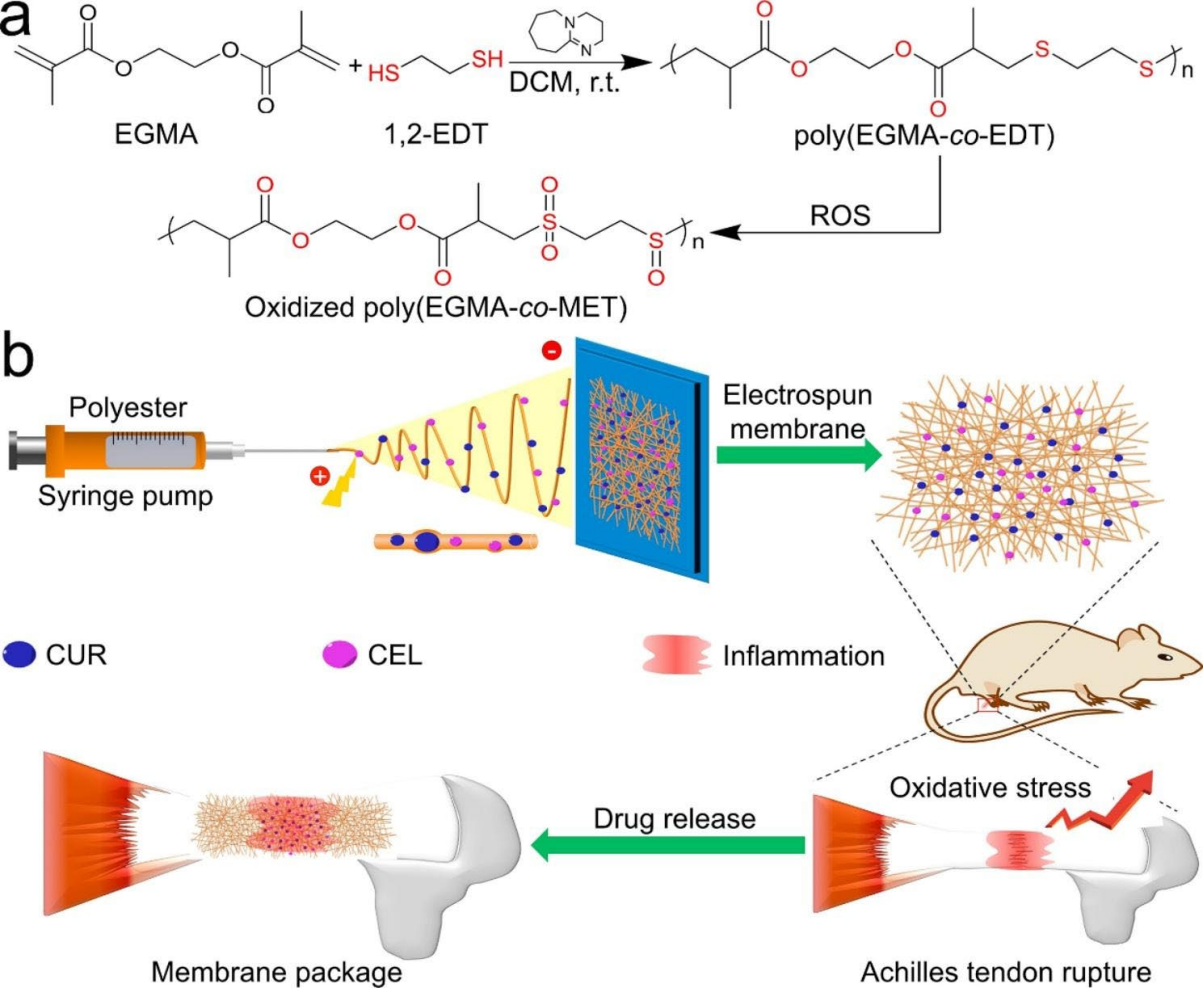
(**a**) Synthesis of ROS-responsive oxidized poly(EGMA-co-EDT). (**b**) Schematic illustrating of preparing drugs-loaded ROS-responsive nanofibers for postoperative tendon anti-adhesion. Reprinted with permission from ref [99]., Elsevier, 2021

**Fig. 3:**
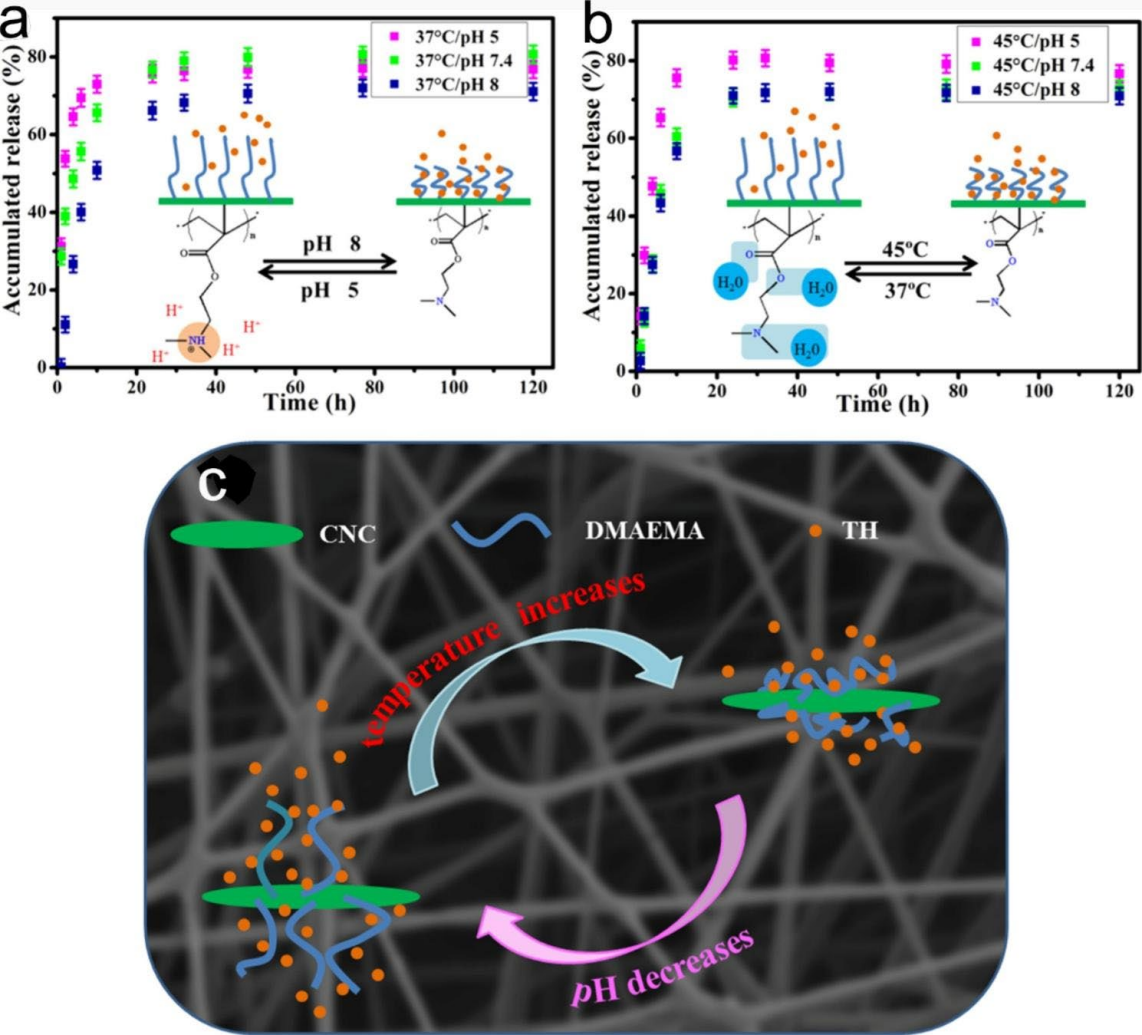
In vitro drug release profiles of double stimuli-responsive cellulose nanocrystals reinforced electrospun PHBV composites membrane at (**a**) 37 °C and (**b**) 45 °C with different pH. (**c**) Schematic diagram of possible drug release mechanism of composite membranes at different physiological pH and temperature. Reprinted with permission from ref [109]., Elsevier, 2020

Electrospun nanofibers have a large specific surface area and high porosity and can simulate the complex three-dimensional micro/nanofiber structure in the extracellular matrix (ECM) to facilitate cell adhesion and proliferation, which is very attractive in the field of biomedicine. More importantly, electrospun nanofibers can efficiently load a variety of drugs (e.g., small molecules, Chinese medicines, proteins, and nucleic acids), and then release the drugs through specific sites, specific doses, and timed to achieve better efficacy and lower toxic side effects. Further, by adding stimulus-responsive materials that can respond to external or internal stimuli, the nanofibers can be complexly regulated to release drugs. At present, the preparation of stimuli-responsive electrospun nanofibers mainly includes blend electrospinning, coaxial electrospinning and post-modification of electrospun nanofibers (Fig. 1b) (Figs. 2, 3).

Blend electrospinning is to add components with specific stimulus response to the spinning solution and form stimuli-responsive nanofibers by electrospinning with a single nozzle [36]. For example, Pham-Nguyen et al. added photosensitizers (chlorin e6, Ce6) to polyoxalates (POX) solution by blend electrospinning to prepare POX/Ce6 nanofibers membrane for cell culture [37]. The nanofibers membrane was excited by the red laser (660 nm) to accelerate the release of ROS and induce the degradation of the nanofibers, thereby promoting the lateral migration of cells. The drug can be directly added to the spinning solution with the stimulus response component to prepare the drug-loaded stimulus response nanofibers by blend electrospinning. However, it is important to note that drugs in nanofibers may burst release, especially water-soluble drugs.

Coaxial electrospinning is to spray two spinning solutions through a coaxial nozzle, and finally form core-shell nanofibers under the action of a high-voltage electric field [38]. The addition of stimuli-responsive substances into the shell can rapidly respond to changes in external conditions. Encapsulating bioactive factors or drugs in the nanofiber core can protect the bioactive factors from being degraded by the in vivo environment and can significantly prolong the drug release time [39]. Zheng et al. fabricated light-responsive nanofibers with a core-shell structure by coaxial electrospinning. Gold nanorods (GNRs) with photothermal properties were uniformly distributed within the shell layer of the nanofibers, and plasmid DNA (pDNA) encoding basic fibroblast growth factor was encapsulated in the core layer. Under near-infrared (NIR) light irradiation, the release of pDNA from the nanofiber cores were increased, and transient pores were created in the membrane of attached cells, facilitating intracellular delivery and pDNA transfection [40].

Stimuli-responsive electrospun nanofibers can also be obtained by post-modification on the surface of nanofibers. Ordinary electrospun nanofibers can immobilize stimuli-responsive substances on the surface of nanofibers through post-modification techniques [41]. Nanofibers surface modification technologies mainly include physical adsorption, layer-by-layer assembly, and chemical immobilization [42]. For example, Schoeller et al. assembled chitosan and sodium alginate layer by layer on PLGA nanofibers to form polyelectrolyte complexes to introduce PH-responsive control of ibuprofen release. Due to the interaction between the drug and the coating, ibuprofen is released more slowly at acidic pH (1.0) than at neutral pH [43].

### Characterization techniques of electrospun nanofibers

Researchers typically utilize a range of techniques to characterize the structure and properties of nanofibers. Scanning electron microscope (SEM) has been widely applied to observe the morphology of nanofibers, including a smooth surface, beaded structure, banded structure, multilayer structure, and rough surface [44]. Furthermore, nanofibers with complicated structures, such as multi-layer coaxial nanofibers and shell-core nanofibers, can be observed by transmission electron microscope (TEM) [45]. For some drugs with fluorescent properties, the distribution of drugs in nanofibers can be observed by fluorescence microscope and confocal laser scanning microscope (CLSM) [38, 46]. The crystal structure and chemical composition of nanofibers can be determined by X-ray diffractometer (XRD) and Fourier transform infrared spectrometer (FTIR) [47]. In addition, nanofibers generally require better mechanical properties when used in tissue engineering, and their mechanical properties can be measured by universal testing machines. For stimuli-responsive electrospun nanofibers loaded with drugs, the study of in vitro release is essential. The release models and kinetics of these drug delivery systems were characterized by varying the stimulus-response conditions in vitro release experiments (e.g., near-infrared light, temperature, magnetic field, electric field, and dissolving media of diverse pH) [10]. These release models and kinetics are important for drug dose prediction in vivo trials.

## Types of stimuli-responsive electrospun nanofibers

Current stimuli-responsive electrospun nanofibers can respond to stimuli including light, temperature, magnetic field, pH, or ROS [10]. Table 1 lists the details of some stimuli-responsive electrospun nanofibers and their applications in the field of biomedicine. Most stimuli-responsive electrospun nanofibers can only respond to one of these stimuli, which limits their application in complex environments. Researchers have developed multiple stimuli-responsive electrospun nanofibers that can respond to two or more stimuli, which can respond to external stimuli more quickly and efficiently, and thus have a wider range of applications [48].

**Table 1:** Stimuli-responsive electrospun nanofibers as intelligent drug delivery platform in biomedicine field

Responsive material	Type of stimulus	Other polymers	Drug	Electrospinning method	Biomedicine	Ref
ICG	NIR light	CS/PVA	Doxorubicin	Blend	Treatment of cervical cancer	[54]
MoS_2_	NIR light	CS/PVA	Doxorubicin	Blend	Inhibit the postoperative tumor reoccurrence	[55]
Pyrrole	NIR light	PCL/PLGA	Doxorubicin	Post-modification	Local cancer treatment following surgical resection	[56]
PNLA	Temperature	PLLA	Rifampicin	Blend	Controllable drug delivery	[57]
Fe_3_O_4_	Magnetic field	PCL/gelatin	Ciprofloxacin	Coaxial	Wound dressing	[58]
ZIF-8	pH	PVA	Vancomycin	Blend	Infected bone repair	[59]
Chi-AO	Electro	PVA	Dexamethasone	Blend	Tissue regeneration	[60]
GO/Au NRs and CS	NIR light/pH	PTMG-PU	Paclitaxel	Coaxial	Treatment of lung cancer	[61]
Iron oxide and Poly(NIPAAm-coHMAAm)	Magnetic field/ Temperature	/	Curcumin	Blend	Treatment of melanoma cancer	[62]
CTS-g-PNIPAAm	Temperature/pH	PEO	BSA	Blend	Drug delivery and tissue engineering	[63]
P(NIPAAm-co-AAc)	Temperature/pH	RSF	Rhodamine B	Blend	Controllable drug delivery	[64]

**Abbreviations**: Au NRs, Gold nanorods; BSA, Bovine serum albumin; Chi-AO, Chitosan-aniline oligomer; CS, Chitosan; CTS-g-PNIPAAm, Chitosan-graft-poly(N-isopropylacrylamide); GO, Graphene oxide; ICG, Indocyanine green; NIR, Near-infrared; P(NIPAAm-co-AAc), poly(N-isopropylacrylamide-co-acrylic acid); PCL, Poly(ε-caprolactone); PEO, Poly(ethylene oxide); PLGA, Poly (D,L-lactic-co-glycolic acid); PLLA, Poly(L-lactide); PNLA, Poly(N-isopropylacrylamide)-b-poly(L-lactide); PTMG-PU, Poly (tetramethylene ether) glycol based-polyurethane; PVA, Poly(vinyl alcohol); Poly(NIPAAm-coHMAAm), Copolymer of N-isopropylacrylamide (NIPAAm) and N-hydroxymethylacrylamide (HMAAm); RSF, Regenerated silk fibroin; ZIF-8, Zeolitic imidazolate framework-8

### Light-responsive electrospun nanofibers

Light-responsive electrospun nanofibers can generate heat or release drugs in response to irradiation at specific wavelengths (ultraviolet, visible, or NIR region), with the possibility of non-invasive and remote spatiotemporal control [49]. The preparation of such stimuli-responsive nanofibers is usually obtained by incorporating light-responsive materials into a polymer matrix [50]. Light-responsive materials mainly include graphene [51], carbon nanotubes [52], indocyanine green [53], black phosphorus [65], gold nanorods [66], and gold nanocages [67]. Li et al. incorporated black phosphorus nanosheets with photothermal properties into electrospun nanofibers. The prepared nanofibers exhibited excellent photothermal properties, and its temperature was increased by 15.26 ℃ under NIR (808 nm, 2.5 W/cm^2^) irradiation, which was able to significantly kill HepG2 cancer cells in vitro [68]. Light-responsive electrospun nanofibers can be loaded with drugs to construct an intelligent drug delivery system to control drug release. For example, Park et al. prepared poly(ε-caprolactone) (PCL) nanofibers loaded with photothermal agent gold nanocages (AuNCs) by electrospinning. The core was loaded with anti-tumor drugs, and the shell was loaded with phase-changeable fatty acid. When irradiated by NIR light, AuNCs generate heat to melt phase-changeable fatty acid, resulting in rapid release of drugs from nanofibers [69].

### Temperature-responsive electrospun nanofibers

Temperature-responsive electrospun nanofibers require the addition of thermosensitive polymers to the nanofibers [70, 71]. Thermosensitive polymers usually have a lower critical solution temperature (LCST) at which they exhibit hydrophilicity, and gradually transition from hydrophilicity to hydrophobicity when the temperature increases beyond their LCST [72]. Poly-N-isopropylacrylamide (PNIPAAm) is the most widely studied temperature-responsive polymer [73]. It is soluble in water at temperatures lower than its LCST (32 °C) and precipitates at higher temperatures. However, PNIPAAm electrospun nanofibers are easily dispersed in water because PNIPAAm is difficult to be cross-linked. Therefore, it can be copolymerized with crosslinkable comonomers to obtain stable nanofibers in aqueous media. Kim et al. polymerized N-isopropylacrylamide (NIPAAm) and crosslinkable N-hydroxymethylacrylamide (HMAAm) and then prepared nanofibers by electrospinning. The hydroxyl groups of HMAAm are then cross-linked by thermal curing. The resulting cross-linked nanofibers exhibited rapid and reversible volume changes with temperature cycling in aqueous media. This dextran-loaded temperature-responsive nanofibers exhibited an “on-off” switchable release of dextran. After six heating cycles, almost glucan was released from the nanofibers, while only a very small amount of glucan was released during cooling [74].

### Magnetic field-responsive electrospun nanofibers

Magnetic field-response electrospun nanofibers move or generate heat when stimulated by a magnetic field [75]. The superparamagnetic nanoparticles are added to the electrospun nanofibers, and these magnetic particles can interact with the external magnetic field to endow the electrospun nanofibers with magnetic field response characteristics [76]. Among various functional superparamagnetic nanoparticles, Co, Fe, Ni, iron oxide and some ferrites are the most popular [77, 78]. Veres et al. prepared polysuccinimide (PSI) nanofibers by electrospinning and crosslinking, and then synthesized magnetic iron oxide nanoparticles (MIONs) inside and between the electrospun nanofibers by co-precipitation. This MIONs-loaded electrospun nanofibers have a remarkable magnetocaloric efficacy, with the temperature of the nanofiber rising over 8 °C within five minutes at a given alternating current magnetic field [79]. Therefore, magnetic field-responsive nanofibers show great potential in hyperthermia-assisted cancer therapy.

### pH-responsive electrospun nanofibers

pH-responsive electrospun nanofibers have a broad prospect in disease treatment because abnormal pH changes in human tissue sites are closely related to pathological conditions [80, 81]. pH-responsive electrospun nanofibers require adding pH-responsive materials to the nanofibers or immobilizing pH-responsive materials on the surface of the nanofibers [82, 83]. pH-responsive materials are usually weak electrolytes, such as alginate [84], chitosan [85], sodium carboxymethyl cellulose [86], polylysine [87], carboxymethyl chitosan [88], polyacrylic acid (PAAc) and so on [89]. Among them, PAAc has a simple structure, obvious pH response and low cost, and is a pH-responsive polymer that has been studied more [90]. For example, Miranda-Calderon et al. prepared three antibiotic-loaded electrospun nanofibers using methacrylic acid copolymer Eudragit® L100-55 (which dissolves at pH > 5.5), Eudragit® S100 (which dissolves at pH > 7) and methacrylic ester copolymer Eudragit® RS100 (which is pH independent). These three pH-responsive wound dressings were able to modulate drug release kinetics in response to changes in the pH of the wound site at different stages of wound healing [91]. In addition, some chemical bonds also have pH-response properties, such as imine bond, 2-propionate 3-methylmaleic anhydride (CDM) bond and phenyl borate bond [92]. For example, the CDM bond is unstable under acidic conditions and breaks. Chen et al. grafted camptothecin (CPT) and α-tocopherol succinate (TOS) onto hyaluronic acid (HA) and then linking the HA conjugates to poly (DL-lactide) (PLA) via CDM bond, prepared short nanofibers by electrospinning. CDM disruption in the weakly acidic microenvironment of tumor tissues (such as pH 6.8) results in the release of HA conjugates and self-assembly into drug-loaded micelles for aggregation to the tumor site, improving therapeutic efficacy and reducing the toxic side effects of chemotherapy drugs [93].

### ROS-responsive electrospun nanofibers

Oxidative stress plays an important role in the pathogenesis of various diseases, including tumors, chronic wounds, myocardial infarction (MI), and postoperative tissue adhesions [94]. During oxidative stress, ROS levels increase dramatically, causing severe damage to cells. Therefore, ROS-responsive electrospun nanofibers are developed to consume ROS or control drug release at specific sites [95]. ROS-responsive nanofibers can be obtained by adding ROS-responsive polymers into the electrospinning solution [96]. ROS-responsive polymers usually have groups such as thioether and ketal in the main or side chain of the polymer [97, 98]. These groups can be cleaved in response to overexpressed ROS in the microenvironment, leading to polymer dissociation and drug release. For example, Zhang et al. synthesized the polymer Poly (ethylene glycol dimethacrylate- co-1,2-ethanedithiol) with ethylene ether groups. The synthetic polymers were then electrospun to prepare ROS-responsive nanofibers loaded with curcumin/celecoxib (CUR/CEL) to prevent peritenendal adhesions. The thioether group in the nanofibers can react with ROS to become hydrophilic sulfoxide or sulfoxide to accelerate the release rate of drugs and intelligently regulate the level of oxidative stress at the inflammatory site (Fig. 2) [99]. Yao et al. synthesized ROS-responsive biodegradable elastic polyurethane containing thioacetone (PUTK) bond, and then the synthetic materials were prepared into glucocorticoid-loaded methylprednisolone electrospun fiber membrane for the treatment of myocardial infarction. The thioketone-containing (PUTK) bond in polyurethane membrane can consume excessive ROS, reduce the damage to heart tissue, and has the potential to prevent and treat cardiovascular diseases. In addition, ROS can trigger the degradation of synthetic polyurethane and accelerate the release of drugs [100].

### Multiple stimuli-responsive nanofibers

Many researchers are designing electrospun nanofibers into multiple stimuli-responsive systems, such as light/temperature [101], pH/ROX [102], pH/temperature [103], light/ magnetic field [104], pH/temperature/ magnetic field [105]. These multiple stimuli-responsive electrospun nanofibers can respond to external stimuli simultaneously or sequentially, and better control drug release through the synergistic interaction between different stimulation behaviors, so as to improve the therapeutic effect [106, 107]. Tiwari et al. modified the surface of doxorubicin (DOX)-loaded PCL nanofibers with multiple stimuli-responsive polydopamine (PDA) to obtain nanofibers with dual pH- and light-responsive properties. Compared with physiological pH conditions (pH 7.4), the acidic medium showed improved drug release. In addition, NIR laser irradiation at 808 nm will lead to a significant increase in local temperature, thus accelerating the release of DOX [108]. This delivery platform is based on dual-responsive properties of pH and light with adjustable drug release properties can be used for local treatment of cancer and other diseases. Some researchers have developed electrospun nanofibers based on the dual response of the internal microenvironment to the specific pH and temperature of certain diseases. In some pathological processes, such as inflammation and tumors, there is a slight increase in local temperature (2–5 °C) or a decrease in pH (1-2.5 pH units). Chen et al. developed pH/temperature responsive electrospun nanofiber membranes as drug delivery carriers based on this feature. The prepared material shows a pH-dependent swelling property and miscibility gap with respect to temperature. When stimulated by internal temperature and pH, the electrospun nanofibers can achieve accelerated drug release at targeted pathological sites, thus providing superior therapeutic effects and less toxic side effects (Fig. 3) [109].

## Application of stimuli-responsive electrospun nanofibers in biomedicine

### Drug delivery

**Fig. 4:**
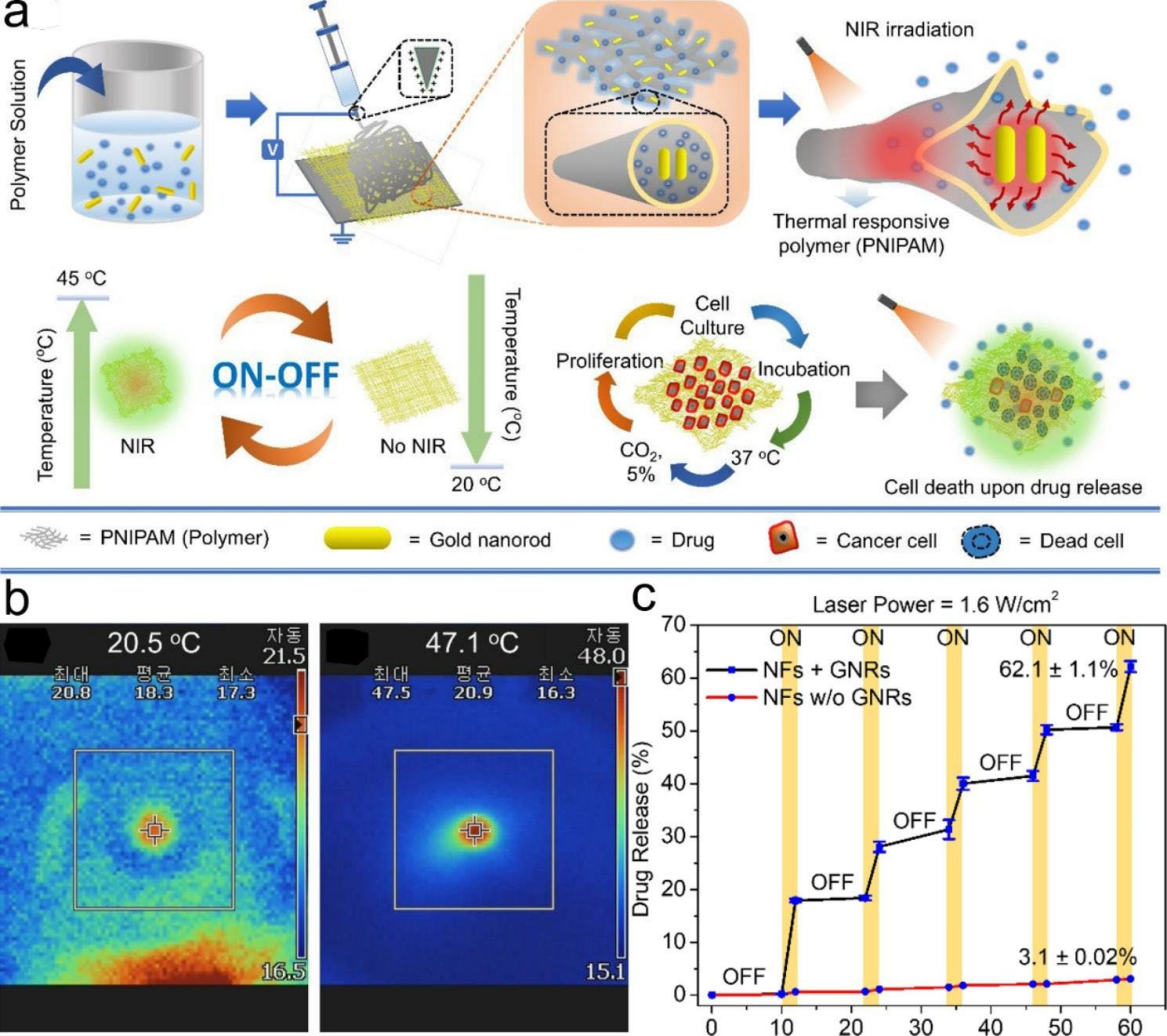
(**a**) Schematic illustration of stimuli-responsive nanofibers containing GNRs for on-demand drug delivery platform. (**b**) Infrared camera images of the nanofibers without GNRs (Left) and with GNRs (Right). (**c**) Pulsatile drug release from the nanofibers through the cyclic on–off of NIR light irradiation at different time intervals. Reprinted with permission from ref [95]., MDPI, 2021

The stimuli-responsive electrospun nanofibers can directly encapsulate drugs in the fibers, which have the characteristics of large loading capacity and high encapsulation efficiency [110, 111]. In addition, they can achieve slow, on-demand or targeted release of drugs under different stimuli [10]. Therefore, stimuli-responsive electrospun nanofibers exhibit great potential for drug delivery. For example, Singh et al. designed poly(N-isopropylacrylamide) (PNIPAM) nanofibers containing gold nanorods (GNRs) as an on-demand drug delivery system. Upon NIR irradiation, the heat generated by GNRs ensures that the nanofibers shrink due to the thermal response of PNIPAM, resulting in the on-off release behavior of the drug (Fig. 4) [112]. Stimuli-responsive electrospun nanofibers can also control the targeted release of drugs at the disease site, thus enhancing the therapeutic effect. For example, the gastrointestinal tract of the human body has a specific pH value, and the release of drugs in different parts of the gastrointestinal tract can be achieved by preparing pH-responsive electrospun nanofibers. Coban et al. prepared niclosamide loaded electrospun nanofibers using pH-responsive polymer (Eudragit®L100). The Eudragit®L100 dissolves at pH values above 6, whereas the colonic pH ranges from 5.7 to 7.7. Therefore, the prepared electrospun nanofibers will not dissolve in the acidic environment of the stomach, and only begin to dissolve and release drugs when they reach the colon, so as to realize the targeted delivery of drugs in the colon [113].

### Cancer therapy

At present, the commonly used clinical cancer treatment methods include surgical resection, chemotherapy, radiotherapy and gene therapy, among which chemotherapy is still the main method for the clinical treatment of various cancers [114–116]. Due to the lack of targeting, chemotherapy can cause damage to normal tissue cells while killing cancer cells, resulting in serious toxic side effects [117, 118]. Therefore, some researchers have developed stimuli-responsive electrospun nanofibers used in cancer treatment, which can respond within the tumor microenvironment of stimulus (such as pH and ROS) or an external stimulus (such as magnetic field and light) to the tumor site targeted release drugs, improve the ability of drugs to kill tumor cells, reduce the harm of human body normal tissue at the same time [119, 120]. Samadzadeh et al. prepared magnetic field-responsive nanofibers by electrospinning a mixture of iron oxide (II, III) magnetic nanoparticles, temperature-responsive copolymers, and mesoporous silica nanoparticles (MSNs) loaded with metformin. Under the action of alternating magnetic field, the nanofibers can be induced to generate heat, promote the release of drugs, thereby increasing the concentration of intracellular drugs and improving the efficacy of chemotherapy. At the same time, hyperthermia can make cancer cells temporarily more sensitive to the destructive effect of anti-cancer drugs, leading to the synergistic effect of magnetic hyperthermia/chemotherapy [121, 122]. Hypoxia and lactic acid accumulation around or within solid tumors lead to decreased pH in tumor tissues, which enables acid-responsive targeted drug delivery in the tumor environment [123, 124]. Yuan et al. loaded sodium bicarbonate (NaHCO_3_) and the anticancer drug doxorubicin (DOX) in mesoporous silica and then blended with polylactic acid (PLLA) to obtain acid-responsive electrospun nanofibers. When nanofibers are delivered to the acidic environment of the tumor, NaHCO_3_ rapidly reacts with acid (H^+^) to produce carbon dioxide gas, and forms a channel inside the fiber to promote the release of DOX, which can effectively kill cancer cells [125].

### Wound dressing

**Fig. 5:**
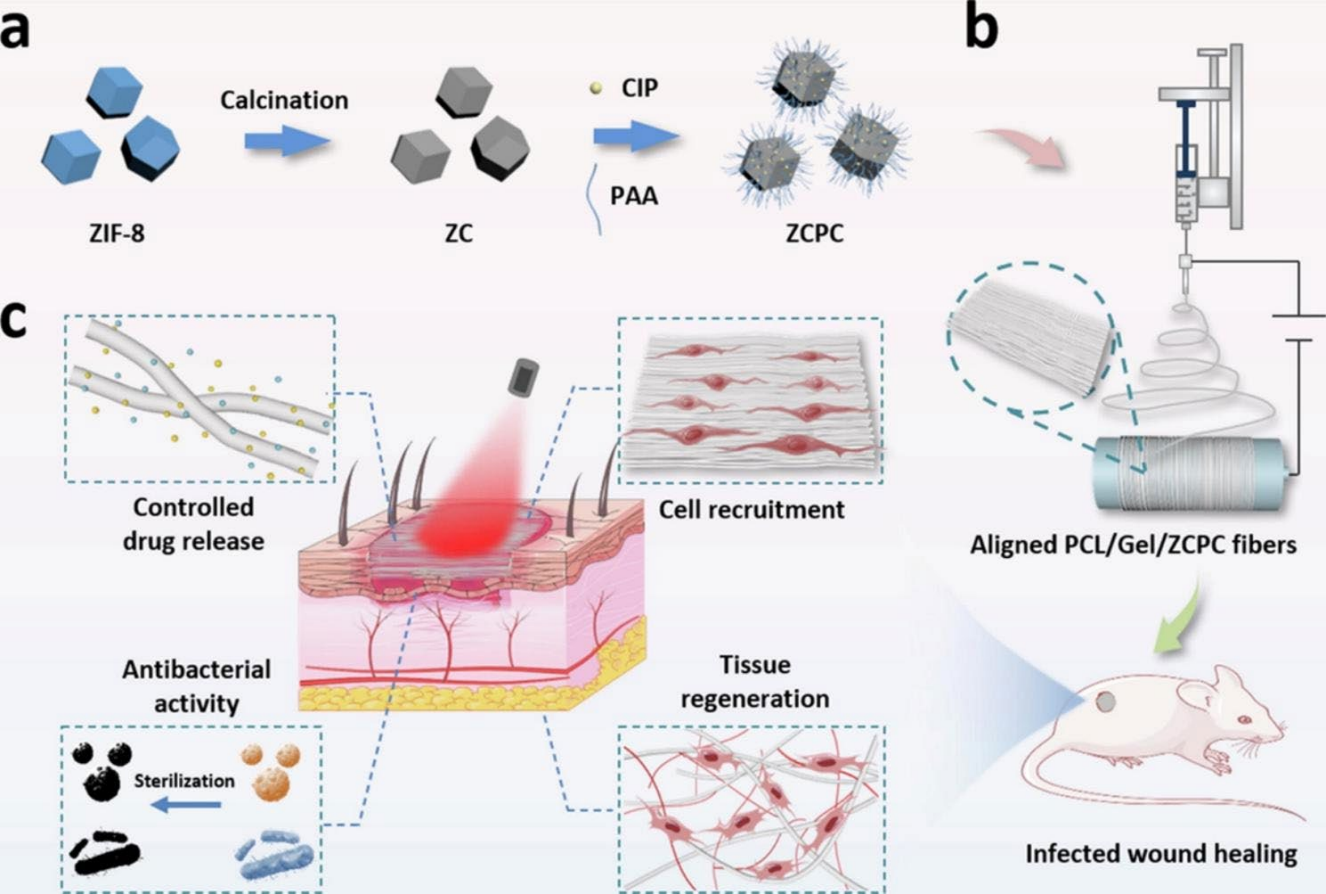
(**a**) Schematic illustration of synthesis of photothermal-chemotherapeutic nanoagent ZCPC. (**b**) Schematic illustration of electrospun aligned PCL/Gel/ZCPC fibers. (**c**) Schematic illustration of the healing mechanism of a full-thickness wound infection model in SD rats. Reprinted with permission from ref [131]., Elsevier, 2022

During wound healing, colonization of the wound site by bacteria and other microorganisms leads to inflammation and delayed healing. While most wound infections will heal on their own, untreated or poorly treated severe wounds can persist and become life-threatening. As a result, many wound dressings with antimicrobial properties have been developed [126, 127]. But there are problems with these dressings, such as premature drug release or poor treatment response due to bacterial resistance [128]. In order to solve these problems, an effective strategy is to develop stimuli-responsive electrospun nanofibers, which can be induced to release a large amount of antimicrobial drugs at a specific time through certain stimuli to improve the antibacterial effect [129, 130]. For example, Chen et al. designed a NIR light-responsive electrospun nanofiber loaded with the antibacterial agent ciprofloxacin hydrochloride (CIP), in which zeolitic imidazolate framework-8 (ZIF-8)-derived nanocarbon were added as the NIR laser trigger. The electrospun fiber membrane generates a lot of heat under near-infrared light irradiation, which accelerates the release of CIP to kill bacteria. The high temperature generated at the same time also destroys the structure of bacterial biofilms, inactivating their active substrates such as nucleic acids and proteins. In addition, it is difficult for bacteria to develop resistance by blocking absorption, increasing metabolism, and drug excretion (Fig. 5) [131]. Photothermally responsive electrospun nanofibers can significantly improve the bactericidal effect through photothermal and bacteriostatic synergistic antibacterial, and avoid the generation of bacterial drug resistance, showing great potential in the field of wound treatment. In addition to infection treatment, real-time monitoring of wound status is also the development direction of stimuli-responsive electrospun nanofibers [132]. Truskewycz et al. synthesized novel fluorescent magnesium hydroxide nanosheets with potent antimicrobial properties, which were then added into PCL/PEO nanofibers as wound dressing. This dressing not only has strong antimicrobial activity, but also has pH-dependent fluorescent properties that can be used to monitor the acidified microenvironment that represents healthy wound healing [133].

### Tissue engineering

**Fig. 6:**
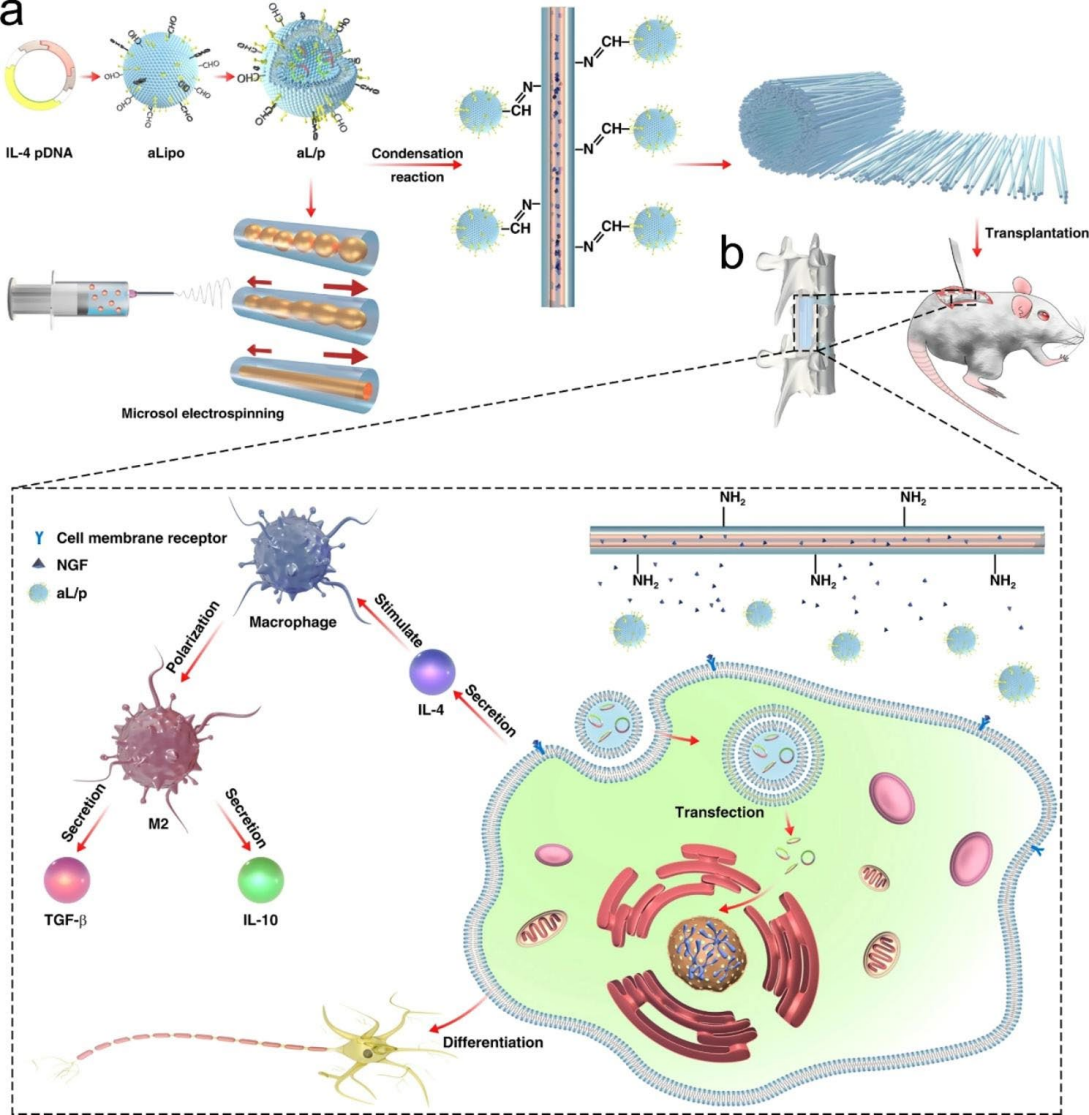
Scheme illustration of (**a**) the construction of bioinspired composite scaffold for the treatment of spinal cord injury along with (**b**) its microenvironment-responsive immune regulation and nerve regeneration effect. Reprinted with permission from ref [135], Springer Nature, 2020

The development of tissue engineering scaffolds is of great significance for the repair and regeneration of damaged tissues or organs. Nanofibers prepared by electrospinning have good mechanical properties, high surface-volume ratio and morphological characteristics similar to ECM, which are widely used for tissue regeneration such as nerves, blood vessels, bone and heart. To enhance the therapeutic effect of electrospun nanofibers, researchers endowed them with intelligent properties to develop stimuli-responsive electrospun nanofibers, which can intelligently release drugs and accelerate tissue regeneration [134]. For example, severe immune inflammation occurs in acute spinal cord injury, which leads to the failure of ordinary electrospun nanofibers to repair nerve tissue. Inspired by the acidic microenvironment at the site of acute spinal cord injury, Xi et al. constructed a functional pH-responsive immunomodulatory electrospun nanofibrous scaffold for assisting nerve regeneration. The stimuli-responsive scaffolds were prepared by grafting the aldehyde-dissociated cationic liposomes loaded with IL-4 plasmid (pDNA) onto the surface of amino-modified directional microsol electrospun nanofibers through Schiff base bonds that would break under acidic conditions. This electrospun nanofiber can directly respond to the local acidic microenvironment and stimulate IL-4 plasmid liposome release in vitro, inhibit the release of inflammatory cytokines, and promote neural differentiation of mesenchymal stem cells, providing an alternative for the treatment of acute spinal cord injury (Fig. 6) [135].

Rapid endothelialization and long-term anticoagulation are a key challenge in the development of tissue-engineered small-diameter vascular grafts [136]. Therefore, it is necessary to add adhesion factors and antithrombotic factors to electrospun nanofiber scaffolds to promote endothelial cell affinity and antithrombotic property. However, the two factors may be released explosively in nanofibers, which cannot achieve a long-term therapeutic effect. Therefore, Wang et al. coated the electrospun nanofibers with gelatin, polylysine and heparin nanoparticles. This coating can be cleaved in response to matrix metalloproteinase-2 dynamically secreted by the vascular extracellular matrix, long-term release of adhesion and antithrombotic factors without burst release, and successfully induce vascular endothelialization and long-term anticoagulation [137].

Some studies have demonstrated that mild photothermal stimulation can effectively promote osteogenesis and bone repair. Therefore, Ma et al. designed PCL/molybdenum disulfide (MoS_2_) nanofibers membrane with photothermal properties for bone regeneration, in which MoS_2_ nanosheets were used as osteogenic enhancer and near-infrared photothermal agent [138]. This nanofibers membrane can enhance cell growth and osteogenic properties. PCL/MoS_2_ nanofibers membrane produced mild photoheat induced by near-infrared laser, which promoted the growth of bone mesenchymal stem cells in vitro, repaired the tibial bone defect in vivo, and promoted osteogenesis and bone healing in rats. This nanofibers membrane can be used as a light-responsive bone scaffold for the treatment of bone defects.

## Clinical status of stimuli-responsive electrospun nanofibers

Stimulation-responsive electrospun nanofibers for drug delivery are a very intriguing and effective way to regulate the release of drugs from the fibers at a specific time or site and are particularly suitable for the treatment of intractable diseases. Compared with systemic drug delivery methods such as intravenous injections, stimulus-response nanofibers can effectively ensure a relatively high drug concentration around local disease tissues, and decrease the distribution of drugs in other normal tissues, thereby reducing the toxic side effects of drugs. Although much work has described the remarkable effects of stimuli-responsive electrospun nanofibers in vitro or in vivo models, they have predominantly remained in the preclinical phase, and data from clinical trials are still very limited. Zhang et al. prepared light-responsive nanofibers loaded with doxorubicin by electrospinning. In vitro cell experiment results showed that the nanofibers could significantly induce cancer cell death. At the same time, the in vivo experiment confirmed that the nanofibers had an obvious inhibitory effect on tumor growth of tumor-bearing mice [139]. While this work demonstrates that stimulus-response nanofibers have good potential for tumor therapy, there are still no clinical trials to confirm the effectiveness of such systems in patients. On the one hand, this drug delivery system requires a substantial amount of external stimulation (for example, electric, magnetic, light, and heat) which can cause damage to healthy tissues. On the other hand, the response parameters are difficult to accurately control and individual differences may lead to a lot of drug leakage and threaten the lives of patients [1]. It has been very challenging to obtain regulatory approval for clinical studies of stimuli-responsive electrospun nanofibers, as their safety and toxicity have not been fully demonstrated. Excitingly, a new electrospun nanofiber scaffold called ReDura™ has been accredited by the China Food and Drug Administration (CFDA) and Conformite Europeenne (CE) for the successful clinical repair of ruptured meninges [140]. Clinical trials of this electrospun nanofiber scaffold can provide clinical data for the development of stimuli-responsive electrospun nanofibers. It is believed that as the safety of stimuli-responsive electrospinning nanofibers is fully verified, they will soon enter the clinical trial stage.

## Conclusions and future perspectives

Electrospun nanofibers can respond to external stimuli at a very high speed due to their great surface area and porosity, making them an ideal stimuli-responsive material. Furthermore, stimuli-responsive nanofibers can efficiently load drugs, and then stimulated by specific conditions (light, temperature, magnetic field, ultrasound, pH or ROS, etc.) to achieve slow, on-demand or targeted release, showing great potential in areas such as drug delivery, tumor therapy, wound dressing, and tissue engineering.

Although several in vitro proof-of-concept of stimuli-responsive electrospun nanofibers have been reported, no stimuli-responsive electrospun nanofibers have made it to the clinic. The toxicity of these smart drug delivery systems is uncertain and is influenced by a variety of factors, including composition, physicochemical properties, route of administration, and dosage. Their composition and drug release mechanisms are more complex than those of ordinary formulations, making their toxicity more difficult to determine accurately. In addition, the clinical efficacy of stimuli-responsive electrospun nanofibers is uncertain, and the difference in response to these agents implanted in each individual may be significant. For example, the depth of the implant site may affect the intensity of light and magnetic field response, and the microenvironment (temperature, pH, and ROS) at the site of the disease is different for different people. It should be noted that the complexity of the architecture design leads to the high difficulty and cost of scale-up production of stimuli-responsive electrospun nanofibers, which limits their development into clinical practice. Ensuring the industrial production and security of electrospun nanofibers in the human body is crucial to promoting their clinical trials. Although stimuli-responsive electrospun nanofibers have great clinical problems, their potential in disease treatment cannot be denied. In particular, stimuli-responsive electrospun nanofibers have unique advantages in targeting intractable diseases such as cancer and tissue repair.

In the future, stimulus-responsive electrospinning nanofibers will evolve in the direction of multi-function and intelligence. For instance, the combination therapy of tumors can inoculate immune cells into stimulus-responsive electrospun nanofibers, and achieve a controlled release of drugs and immune cells at the same time after implantation in the body to jointly kill tumor cells [141]. In addition, disease diagnostic imaging technology and stimulus-responsive electrospinning nanofiber drug delivery systems are combined to enable precision therapy by monitoring pathological processes in deep tissues and then triggering the quantitative release of drugs [142]. In conclusion, stimuli-responsive electrospun nanofibers may be a promising drug delivery strategy to control drug release in response to different stimuli, especially disease-specific signals, but their clinical application is still a long way off.

## Data Availability

Not applicable.
